# Heavy-ion beam-induced mutants of *Medakamo hakoo* indicate potential associations between photosynthesis and cell size, cell cycle, and cell wall morphology

**DOI:** 10.1007/s10265-025-01680-2

**Published:** 2025-12-10

**Authors:** Yoji Okabe, Yayoi Tsujimoto-Inui, Shinichiro Maruyama, Kazuhide Tsuneizumi, Tsuyoshi Takeshita, Mayuko Sato, Kiminori Toyooka, Tomoko Abe, Sachihiro Matsunaga

**Affiliations:** 1 Graduate School of Frontier Sciences, The University of Tokyo, Chiba, Japan; 2 RIKEN Nishina Center for Accelerator-Based Science, Saitama, Japan; 3 Graduate School of Human and Environmental Studies, Kyoto University, Kyoto, Japan; 4 RIKEN Center for Sustainable Resource Science, Yokohama, Kanagawa Japan

**Keywords:** Cell cycle, Cell wall, Green algae, Heavy-ion beam, *Medakamo hakoo*, Photosynthesis

## Abstract

*Medakamo hakoo* is an ultrasmall green alga with a simplified cellular structure, offering potential as a new model organism. To explore the genetic basis of cell morphology and its physiological implications, we applied carbon-ion beam irradiation to induce mutations in *M. hakoo* and successfully isolated two mutants: *LRG* (Large) and *TTR* (Tetra). *LRG* exhibited significantly enlarged cell size and increased chlorophyll content, but lower photosynthetic efficiency compared to the wild-type. Proteomic analysis of *LRG* revealed upregulation of photosynthesis-related proteins, stress response proteins, and ribosomal proteins, suggesting a link between increased cell size and disrupted photosynthetic homeostasis. *TTR* showed a higher proportion of dividing cells throughout the light–dark cycle and exhibited cell aggregation. Proteomic profiling revealed increased abundance of dynein and cell wall–modifying enzymes such as expansin and mannosidases, indicating that *TTR* may undergo cell cycle delay or dysregulation of cytoplasmic and cell wall dynamics. *TTR* also displayed decreased levels of photosynthetic proteins and reduced photosynthetic activity. Both mutants demonstrated slower growth compared to the wild-type. These findings highlight the close relationship between cell size and cell cycle/cell wall dynamics with photosynthetic activity, providing new insights into the cellular regulatory mechanisms of photosynthetic microalgae. Our study also demonstrates the utility of heavy-ion mutagenesis in dissecting microalgal physiology in species for which genetic transformation systems have not yet been developed.

**Supplementary Information:**

The online version contains supplementary material available at 10.1007/s10265-025-01680-2.

## Introduction

Algae represents one of the most evolutionarily diverse groups of photosynthetic organisms, thriving in a wide range of terrestrial and aquatic habitats including extreme environments characterized by high salinity, low pH, and low temperature (Lewis and McCourt 2004). Among them, green algae are regarded as the closest relatives of land plants, together forming the monophyletic group Viridiplantae (Bachy et al. 2022; Lewis and McCourt 2004). Elucidating the adaptation strategies of green algae through physiological and cell biological approaches, thereby revealing both aspects that share basic mechanisms with land plants and aspects that have evolved in a lineage-specific manner, is expected to provide valuable insights to plant biology as a whole. The unicellular green algae *Medakamo hakoo* Kuroiwa has the smallest cell size (1–2 μm), genome (15.8 Mbp), number of genes (7629 genes), and only a single mitochondrion and chloroplast (Kato et al. 2023; Kuroiwa et al. 2015, 2016; Takusagawa et al. 2023). These features suggest that *M. hakoo* represents a highly simplified cell, making a promising candidate as a model organism for green algae (Goold et al. 2024). However, a stable transformation system for *M. hakoo* has not yet been established, which currently hinders the application of reverse genetic approaches.

To advance research on *M. hakoo*, we employed heavy-ion beam irradiation as a means of mutagenesis. This method induces random genomic mutations and, owing to its high linear energy transfer (LET), directly damages DNA by causing double-strand breaks. As a result, it generates a broader and more diverse spectrum of mutations compared to gamma rays, X-rays, or other chemical mutagens　(Abe et al. 2015; Guo et al. 2024). Importantly, heavy-ion beam irradiation enables mutant generation without the need for transformation techniques. Due to its effectiveness, heavy-ion beam irradiation has been widely applied to generate mutant strains in various algal species. Its utility in algal research has already been demonstrated in multiple studies. However, most of these efforts have focused on enhancing the production of industrially relevant compounds such as carotenoids and biofuels (Ma et al. 2013; Takeshita et al. 2018, 2021). In contrast, relatively few studies have investigated mutations that affect cellular morphology including cell size and shape for their biological significance. In this study, to obtain mutant strains exhibiting altered cell size and shape, we irradiated *M. hakoo* with heavy-ion beam. As a result, we successfully isolated two mutants and conducted phenotypic analyses to characterize their cellular features.

## Materials and methods

### Cultivation of algal strains

Wild-type and mutant strains of *M. hakoo* were maintained in either MGRL medium (Fujiwara et al. 1992) or AF-6 medium (NIES). During the period from beam irradiation to mutant selection, cultures were grown in MGRL medium. Subsequently, the medium was switched to AF-6 medium upon observation that *M. hakoo* exhibited enhanced growth in AF-6.

For liquid culture, algal cells were inoculated into 10–30 mL of liquid medium in 25-mL flasks (VTC-F25V, AS ONE) and incubated at 25 °C under continuous shaking at 105 rpm. Illumination was provided at 100 μmol m⁻^2^ s⁻^1^ with a 12 h light/12 h dark photoperiod. For plate culture, cells were spread onto MGRL medium solidified with 1.5% (w/v) gellan gum and incubated at 22 °C under 80 μmol m⁻^2^ s⁻^1^ light intensity with a 16 h light/8 h dark cycle.

### Heavy-ion beam irradiation and mortality measurement

Algal cells maintained on MGRL solid medium were transferred to MGRL liquid medium and cultured for one week at 22 °C under 80 μmol m⁻^2^ s⁻^1^ illumination with a 16 h light/8 h dark photoperiod. Cells in the exponential growth phase, with an optical density (OD₇₅₀) of approximately 0.7, were aliquoted into PCR tubes (200 μL per tube). These samples were irradiated with a carbon-ion beam (LET: 23 keV μm^−1^) at doses of 0, 12.5, 25, 50, 100, and 150 Gy at the RI-Beam Factory (RIBF) in RIKEN (Wako, Saitama, Japan).

Following irradiation, the cells were kept in the dark for at least 3 h. Subsequently, 500 cells from each treatment group were plated onto MGRL medium solidified with gellan gum and incubated until visible colonies formed. Colony numbers were counted after 26 days of incubation (*n* = 3). The colony mortality rate was calculated using the following formula:

$$\begin{aligned}{\text{Colony mortality rate}(\%)}&= \left(1 - \frac{\text{Average number of colonies at each Gy}}{\text{Average number of  colonies at 0 Gy}} \right)\times 100\end{aligned}$$

Colonies from the mortality assay plates were transferred to fresh MGRL plates for further screening of mutant strains. For subsequent analyses, selected strains were maintained in AF-6 liquid medium.

### Screening mutants by microscopic observations

Each mutant strain cultured in liquid medium was centrifuged at 10,000 × *g* for 3 min. After removing most of the supernatant, approximately 3 μL of the cell pellet was placed onto a glass slide and covered with a cover slip. The sample was allowed to rest for 10 min to minimize cell movement prior to observation. Microscopic examination was performed using an upright microscope (BX53, Evident).

#### Electron microscopy

Cells in the exponential growth phase were mixed with an equal volume of fixative solution (1% glutaraldehyde in 0.02 M sodium cacodylate). Fixation was carried out in the dark at 4 °C for more than 24 h. The samples were then sent to RIKEN CSRS (Yokohama, Kanagawa, Japan), washed with 0.02 M sodium cacodylate, and post-fixed for 2 h in 1% osmium tetroxide in 0.02 M sodium cacodylate. After washing with distilled water, the samples were dehydrated through a graded ethanol series followed by propylene oxide, and embedded in Spurr’s resin. Polymerization was performed at 70 °C for 24 h. Ultrathin sections (70–80 nm thick) were cut, stained with uranyl acetate and lead citrate, and observed under a JEM-1400 Flash transmission electron microscope operated at 80 kV (JEOL, Tokyo, Japan).

### Cell size measurement

Microscopic images of over 250 cells per replicate from wild-type and mutants in the exponential growth phase were captured. Cell size was quantified using the “Analyze Particles” function in Fiji software (Schindelin et al. 2012), which automatically measured the area of each cell in the images.

### Chlorophyll quantification

Chlorophyll content was determined by methanol extraction followed by spectrophotometric analysis. One milliliter of cells in the exponential growth phase was collected into a microtube and centrifuged at 10,000 × *g* for 5 min to remove the supernatant. The pellet was resuspended in 1 mL of 100% methanol (FUJIFILM Wako) by pipetting and vortexed for 10 s. The sample was incubated in the dark at 4 °C for approximately 24 h, then centrifuged again at 10,000 × *g* for 10 min. The supernatant was transferred to a new tube, and an additional 1 mL of 100% methanol was added. The entire volume was transferred to a glass cuvette, and absorbance was measured at 652, 665, and 750 nm using a spectrophotometer.

Chlorophyll concentration (μg mL^−1^) was calculated according to the method described by Porra et al. (1989). Cell concentration was estimated from the initial OD₇₅₀ of the culture, and chlorophyll content per cell was derived accordingly.

### Counting of dividing cells

Cells cultured under a 12 h light/12 h dark cycle were sampled at 6 h after the onset of the light phase and 4 h after the onset of the dark phase, corresponding to the division phases described by Kato et al. (2023). The cell suspension was centrifuged at 10,000 × *g* for 3 min to remove the supernatant. An equal volume of fixative solution (1% glutaraldehyde in 0.02 M sodium cacodylate) was added, and the sample was vortexed for 3 s. Cells were fixed in the dark at 4 °C for over 20 h.

After fixation, the solution was replaced with phosphate-buffered saline (PBS). The sample was vortexed briefly (1 s) and incubated in the dark for 10 min. Brightfield and chlorophyll fluorescence images were acquired using a confocal laser scanning microscope (FLUOVIEW FV1200, Evident).

Cells were classified into division stages—singlet, doublet, and tetrad—based on criteria described by Kato et al. (2023). Quantification was performed using the “Analyze Particles” function in Fiji software, supplemented by manual visual counting.

### Proteome analysis

Cells in the exponential growth phase were harvested during the light phase (6 h after the onset of illumination) and centrifuged at 10,000 × *g* for 3 min to remove the supernatant. The resulting pellet was flash-frozen in liquid nitrogen and stored at − 80 °C for 1–2 days. Total protein was extracted using the Minute Detergent-Free Protein Extraction Kit for Microbes with Thick Cell Walls (YD-016, Invent Biotechnologies), following the manufacturer’s protocol with the exception of the grinding step. Instead of the kit’s recommended method, samples were ground using a BioMasher II (893,063, Nippi) in combination with a PowerMasher II (893,002, Nippi).

For the initial grinding step, 50 μL of buffer A and 80–90 mg of protein extraction powder were added to the sample and ground for 10 s with 10-s intervals on ice, repeated three times. Subsequently, 50 μL of buffer A and 100 μL of buffer B were added, and the grinding procedure was repeated under the same conditions. All subsequent steps followed the kit protocol. Protein concentration was determined using the Bio-Rad Protein Assay Kit II (5,000,002, Bio-Rad), and samples were stored at − 80 °C until mass spectrometry analysis.

Proteomic analysis was performed using Orbitrap Exploris 480 mass spectrometer (Thermo Fisher Scientific) at Nagoya University (Nagoya, Aichi, Japan). MS/MS spectra were processed and peak lists generated using Proteome Discoverer version 2.2.0.388 (Thermo Fisher Scientific). Peptide identification was conducted using the SEQUEST algorithm against an in-house *X* protein database with the following parameters: enzyme specificity allowing up to two missed cleavages; peptide mass tolerance of 10 ppm; MS/MS tolerance of 0.02 Da; fixed modification of carbamidomethylation (C); and variable modification of oxidation (M). Peptides were identified based on significant Xcorr values (high-confidence filter). Identified peptides and their modifications were manually reviewed and filtered to generate a confirmed list for higher-energy collisional dissociation (HCD) MS/MS analysis. Label-free quantification was performed using precursor ion intensities normalized to the total peptide amount.

Normalized abundance values for each protein were calculated by dividing the abundance of each replicate (*n* = 3) by the total abundance of each replicate. These normalized values were log₂-transformed and subjected to differential expression analysis using the limma package (Ritchie et al. 2015). Proteins with a log₂ fold change > 0 and a false discovery rate (FDR) < 0.1 (Benjamini–Hochberg correction) were considered significantly more abundant, while those with a log₂ fold change < 0 and FDR < 0.1 were considered significantly less abundant. These proteins were used for subsequent analyses.

### Measurement of photosynthetic parameters

Photosynthetic parameters (Fv/Fm, Φ_Ⅱ_, Φ_NPQ_, Φ_NO_) were measured using the microscopy version of the IMAGING-PAM system (Walz). Cultures in the exponential growth phase were used for analysis. A 3 μL aliquot of cell suspension was placed onto a glass slide and covered with a cover slip. The slide was positioned on the microscope stage and focused using a 10 × objective lens (Objective Fluar 10 × /0.5 M27, Zeiss).

Highly concentrated regions of cells near the edge of the cover slip were selected within the field of view. Ten areas of interest (AOIs) were defined using Imaging WinGigE software (Walz). After a 5-min dark adaptation under 470 nm pulse-modulated measuring light, the minimum fluorescence (F_0_), fluorescence (F) and the maximum fluorescence (Fm, Fm′) were recorded for each AOI by applying saturating pulses under continuous actinic light (0–177.7 μmol m⁻^2^ s⁻^1^).

The maximum quantum yield of PSⅡ (Fv/Fm) was calculated using the following equation:

$${\mathrm{Fv}}/{\text{Fm }} = \, \left( {{\mathrm{Fm}} - {\mathrm{F}}_{0} } \right)/{\text{ Fm}} $$

The effective quantum yield of photosystem II (Φ_Ⅱ_) was calculated using the following equation:

$$\Phi_{\mathrm{II}} = \, \left( {{\mathrm{Fm}}^{\prime } - {\mathrm{F}}} \right)/{\text{ Fm}}^{\prime } $$

The quantum yields of regulated (Φ_NPQ_) and nonregulated (Φ_NO_) energy dissipation in PSII were calculated according to Kramer et al. (2004) using the following equations:

$$\Phi_{\mathrm{NPQ}} = {1}{-}\Phi_{\text{II }} - {1}/\left( {{\mathrm{NPQ}} + {1} + {\mathrm{q}_{L}}\left( {{\mathrm{Fm}}/{\mathrm{F}}_{0} - {1}} \right)} \right) $$

$$\Phi_{\mathrm{NO}} = {1}/\left( {{\mathrm{NPQ}} + {1} + {\mathrm{q}_{L}}\left( {{\mathrm{Fm}}/{\mathrm{F}}_{0} - {1}} \right)} \right) $$

In these equations, NPQ and q_L_ were calculated using the following equations:

$${\mathrm{NPQ}} = \left( {{\mathrm{Fm}} - {\mathrm{Fm}}^{\prime } } \right)/{\text{ Fm}}^{\prime } $$

$${\mathrm{q}_{L}} = \left( {{\mathrm{Fm}}^{\prime } - {\mathrm{F}}} \right)/\left( {{\mathrm{Fm}}^{\prime } - {\mathrm{F}}_{0}^{\prime } } \right)\times{\mathrm{F}}_{0}^{\prime } /{\mathrm{F}} $$

Besides, F_0_′ (minimal fluorescence yield of illuminated sample) in q_L_ equation was estimated using the following equation according to Oxborough and Baker (1997):

$${\mathrm{F}}_{0}^{\prime } = {\mathrm{F}}_{0}/ \, ({\mathrm{Fv}}/{\mathrm{Fm}} + {\mathrm{F}}_{0}/{\mathrm{Fm}}^{\prime } ) $$

### Growth measurement

Cultures were adjusted to an optical density at 750 nm (OD₇₅₀) of approximately 0.1 and inoculated into 20 mL of AF-6 medium. Incubation was performed under the conditions described above. OD₇₅₀ was measured every 1–3 days using a spectrophotometer (NanoPhotometer Pearl, Implen).

To correlate OD₇₅₀ with cell concentration, a dilution series of cultures (OD₇₅₀ = 0.1–2.0) was prepared. Cell concentrations were determined using a cell counting plate (CP-BT, Ina-Optika), and a calibration curve was generated to relate OD₇₅₀ values to cell numbers. OD₇₅₀ measurements obtained during growth monitoring were converted to cell concentrations using this calibration curve.

### Statistical analysis and graphing

All statistical analyses and data visualizations were performed using R software (version 4.4.1).

## Results

### Isolation of mutants of *M. hakoo* by irradiation of carbon-ion beam

**Fig. 1:**
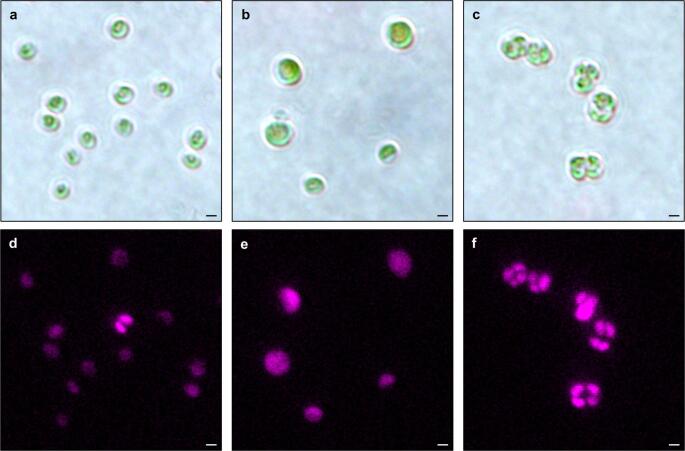
Wild-type (WT) and two mutants of *M. hakoo* generated by heavy-ion beam irradiation. **a**–**c** Bright- field images; **d**–**f** chlorophyll fluorescence images. **a**, **d** WT; **b**, **e** *LRG*; **c**, **f** *TTR*. Scale bars: 1 μm

To determine the optimal irradiation conditions for generating *M. hakoo* mutant strains, we first evaluated the colony mortality rate following carbon-ion beam irradiation. Mortality increased with irradiation dose; however, even at high doses (100 and 150 Gy), the average mortality rate remained below 80% (66.6% on average at 100 Gy, 78.6% on average at 150 Gy). Since *M. hakoo* retained colony-forming ability under high-dose irradiation, we collected approximately 1,500 colonies that had formed after exposure to each dose (12.5, 25, 50, 100 and 150 Gy). Next, cells derived from these colonies were observed under a microscope, and two mutant strains exhibiting distinct morphological features were isolated (Fig. 1). The mutant obtained from 100 Gy irradiation exhibited larger cells compared to wild-type (WT) and was designated as “Large” (*LRG*). The mutant derived from 150 Gy irradiation displayed an increased number of cells resembling those in the four-cell division stage and was designated as “Tetra” (*TTR*). Based on these unique phenotypes, we selected these two mutants for further characterization.

### The intracellular structures of the mutants

**Fig. 2:**
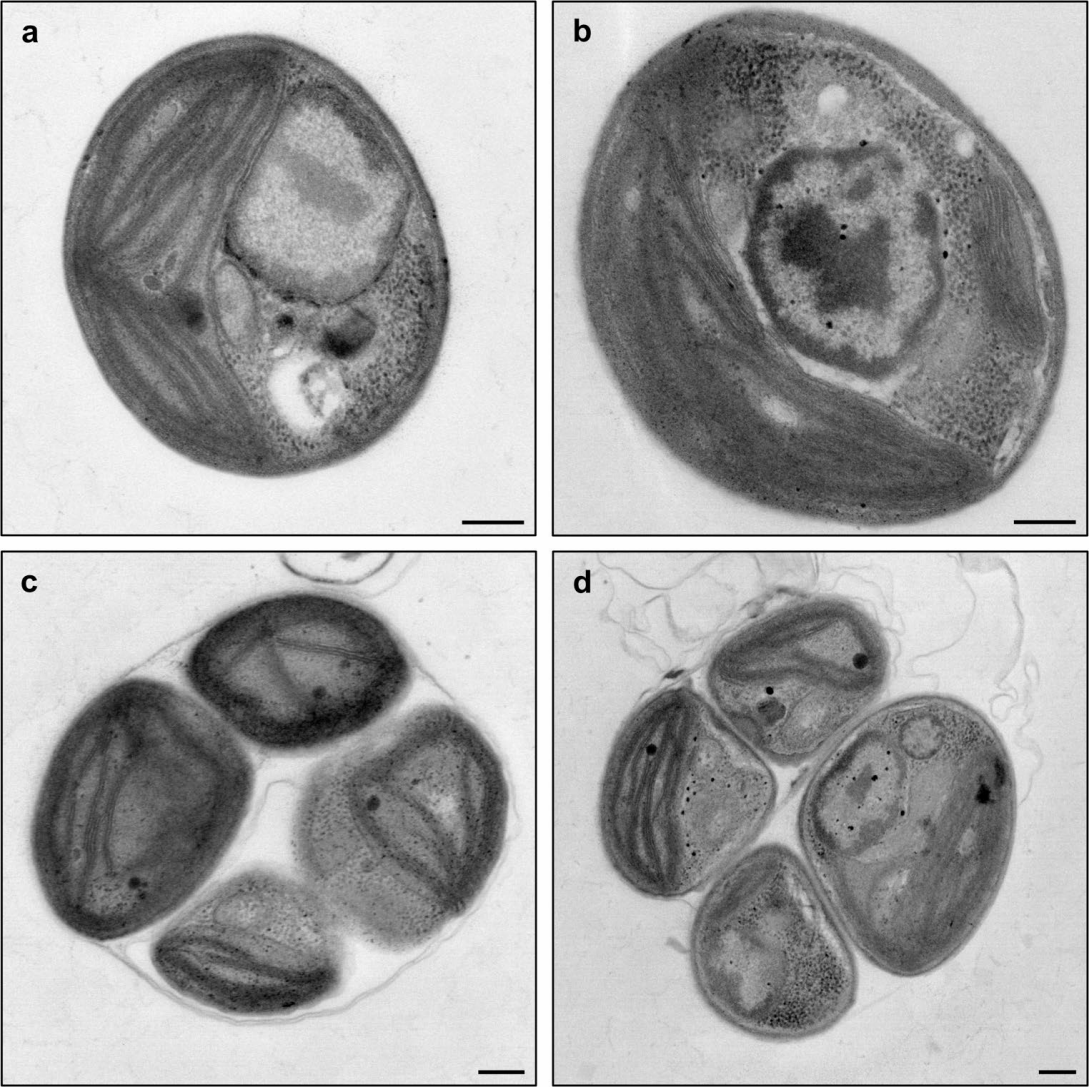
Transmission electron micrographs of the mutants. **a**, **c** WT at the one-cell and four-cell stages. **b** *LRG* at the one-cell stage. **d** *TTR* at the four-cell stage. Scale bars: 200 nm

To examine the detailed cellular structures of the mutants, transmission electron microscopy (TEM) was performed (Fig. 2). Compared with WT, the *LRG* mutant exhibited uniformly enlarged organelles such as the nucleus and chloroplast, without marked alterations in their relative proportions or overall morphology (Fig. 2a, b). In the *TTR* mutant, many cells displayed cellular structures similar to those of WT at the four-cell stage (Fig. 2c, d). However, some cells showed deformed cell walls and aggregation (Fig. S1).

### *LRG* showed enlarged cells and high amount of chlorophyll

**Fig. 3:**
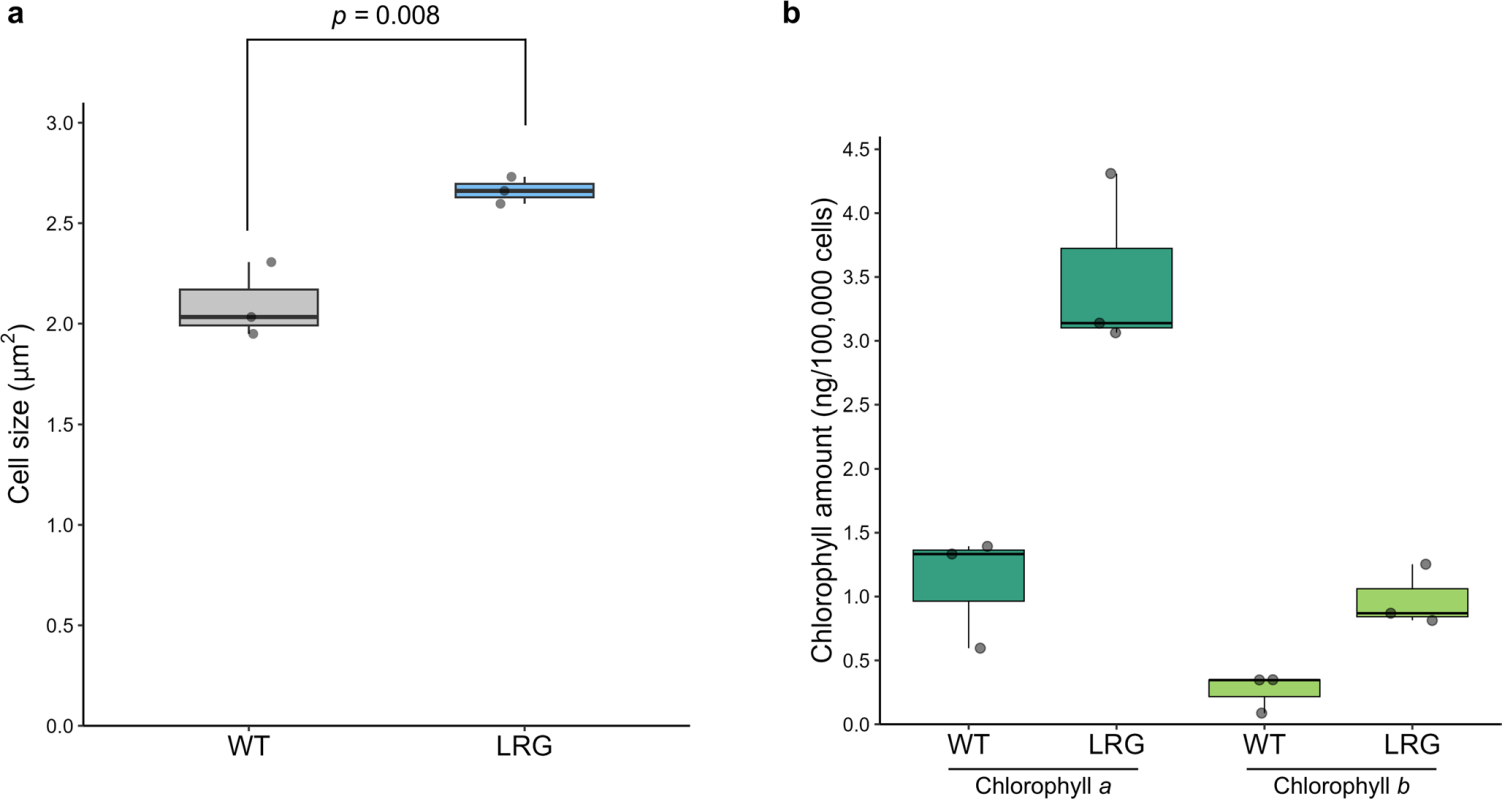
Phenotypic characteristics of *LRG* strain. **a** Cell size. Each dot represents the average cell size of an individual replicate (*n* = 3). The *p* value was calculated using Student’s t-test. **b** Chlorophyll *a* and *b* content per cell. Each dot represents the chlorophyll amount of an individual replicate (*n* = 3)

We first investigated the cell size of the *LRG* mutant (Fig. 3a). On average, *LRG* cells were 27.1% larger than those of WT (WT: 2.10 μm^2^ in average; *LRG*: 2.66 μm^2^). Since the color of the culture medium between WT and *LRG* at the same cell concentration was different, we quantified the amount of chlorophyll per 100,000 cells (Fig. 3b). *LRG* contained 3.2 times more chlorophyll *a* than the WT (WT: 1.1 ng in average, *LRG*: 3.5 ng in average). Chlorophyll b was also 3.8 times higher in *LRG* (WT: 0.26 ng in average, *LRG*: 1.0 ng in average). When total chlorophyll content (chlorophyll *a* and *b*) was compared, *LRG* exhibited 3.3 times higher levels than WT (WT: 1.4 ng in average, *LRG*: 4.5 ng in average).

### *TTR* exhibited a high proportion of dividing-like cells and showed cell aggregation

**Fig. 4:**
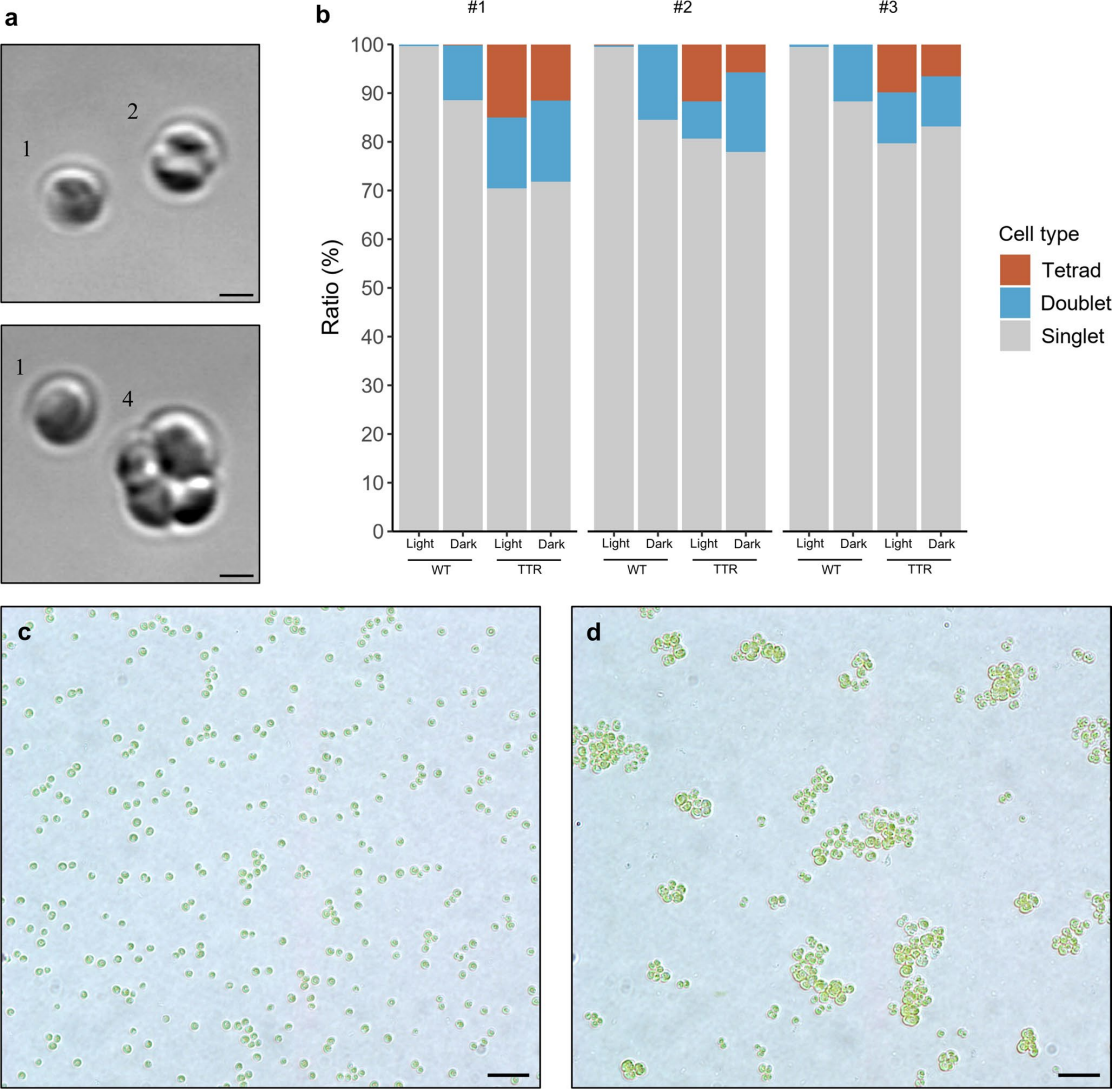
Proportion of dividing cells and cell aggregation in the *TTR* mutant. **a** Micrographs of mitotic cells in WT. 1, singlet; 2, doublet; 4, tetrad. Scale bars: 1 μm. **b** Proportion of dividing cells in the light and dark phases. Light, 6 h after the start of the light phase; Dark, 4 h after the start of the dark phase. #1, #2, and #3 represent independent replicates cultured in different flasks. **c** Microscopic image of WT. **d** Microscopic image of the *TTR* mutant showing cell aggregation. Scale bars: 10 μm

Since the *TTR* strain frequently exhibited four-cell–like dividing stages, we hypothesized that its proportion of dividing cells during the light–dark cycle might differ from that of WT. To test this, we quantified the ratios of dividing-like cells (singlets, doublets, and tetrads) at two time points: 6 h after the onset of the light phase and 4 h after the onset of the dark phase (Fig. 4a, b). In WT, the proportion of dividing cells (doublets and tetrads) ranged from 0.3 to 0.5% during the light phase and increased to 11.5–15.5% in the dark phase. In contrast, *TTR* showed a markedly higher proportion of dividing cells in both phases, ranging from 19.4 to 29.6% in the light phase and 16.8–28.2% in the dark phase. Moreover, *TTR* consistently formed cell aggregates regardless of the light–dark cycle (Fig. 4c, d).

### Proteomic analysis of *LRG* mutant reveals alterations in chloroplast, stress response, and ribosomal proteins

**Fig. 5:**
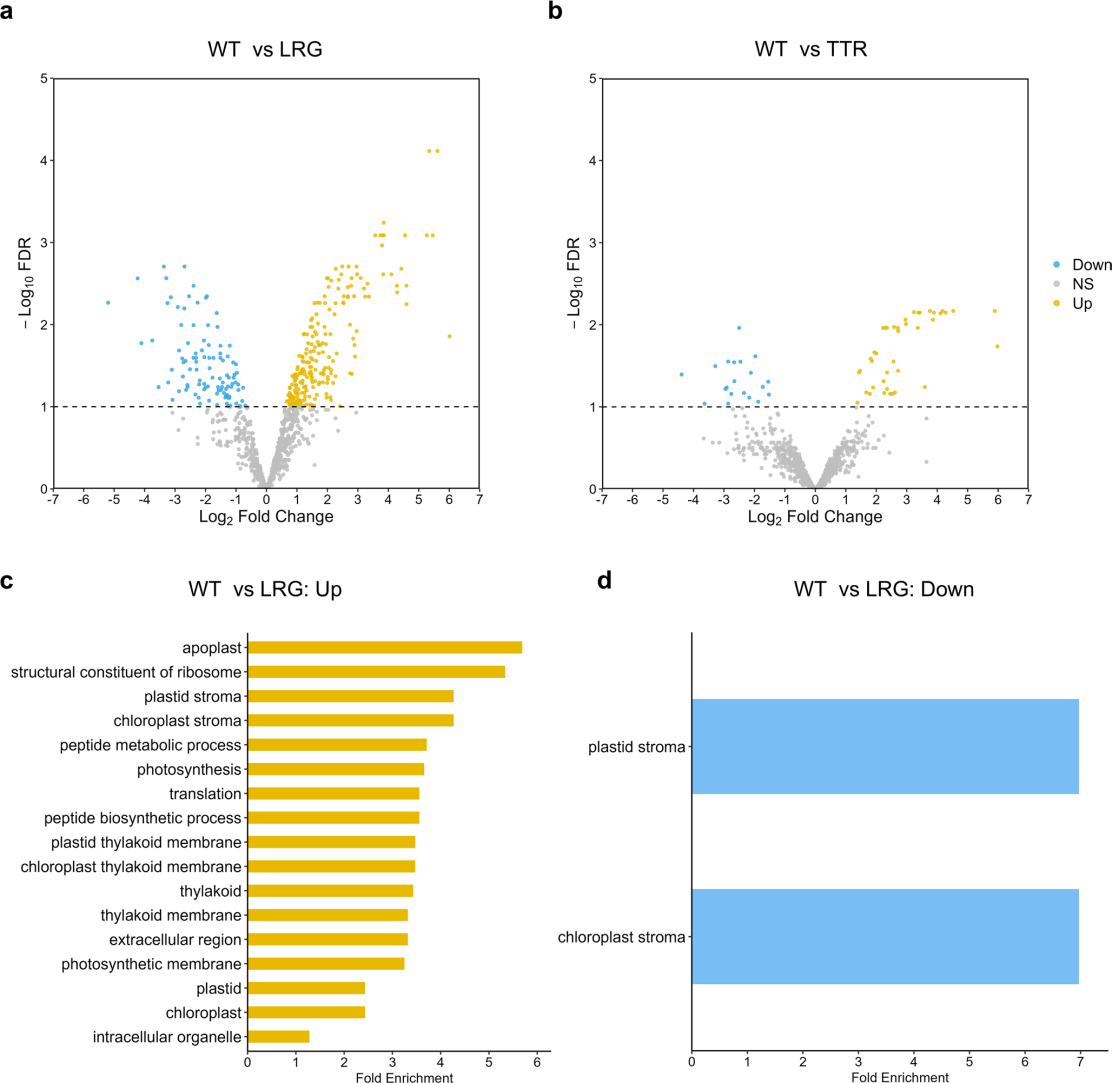
Proteomic changes of the mutants. **a**, **b** Volcano plots showing differential protein abundance in mutants compared with WT (*n* = 3). The horizontal dotted line indicates a false discovery rate (FDR) threshold of 0.1. Up: the proteins significantly more abundant in the mutant than WT (FDR < 0.1). Down: the proteins significantly less abundant in the mutant. NS: not significantly abundant. **c**, **d** Significantly enriched GO-terms of the proteins in *LRG* (*n* = 3, FDR < 0.1)

To identify proteins potentially responsible for the observed mutant phenotypes, the proteome analysis was performed across the growth phases of the mutants. As a result, a total of 1,058 proteins were detected. Among these, the abundances of 322 proteins were significantly altered in *LRG* compared with WT (Fig. 5a. more abundant: 221 proteins, less abundant: 101 proteins, FDR < 0.1). In *TTR,* the abundances of 61 proteins were significantly changed (Fig. 5b; increased in 41 proteins, decreased in 20 proteins).

To further investigate the functional implications of these changes, de novo GO analysis was conducted on the differentially abundant proteins. Because *M. hakoo* proteins are not yet annotated in public GO databases, we manually generated a reference GO annotation set using eggNOG-mapper (Cantalapiedra et al. 2021) based on the nuclear genome-derived protein sequences of *M. hakoo*. The resulting annotations were provided in Table S1.

After constructing the annotation reference, GO enrichment analysis was performed using clusterProfiler package (Yu et al. 2012). As a result, several GO terms were significantly enriched among the proteins that were either more or less abundant in *LRG* (Fig. 5c, d). In the group of more abundant proteins, many GO terms associated with the chloroplast were enriched, including “chloroplast”, “chloroplast stroma”, “thylakoid”, and “photosynthesis” (Fig. 5c). In the group of less abundant proteins, two GO terms, “plastid stroma” and “chloroplast stroma”, were also enriched. Notably, these terms overlapped with the more abundant group, but the proteins involved were distinct (Fig. 5d). To further explore which specific proteins were differentially abundant in *LRG*, we mapped protein IDs with KEGG K numbers (Kanehisa and Goto 2000), using the previously generated eggNOG annotation as a reference. As a result, 184 proteins in *LRG* were assigned K numbers (133 more abundant and 51 less abundant; Tables S2 and S3). Among the more abundant proteins, we identified numerous photosynthesis-related proteins, such as the chlorophyll synthases: geranylgeranyl diphosphate reductase (ChlP), the components of photochemical complex: photosystem II oxygen-evolving enhancer protein 2 (PsbP; two peptides, MHACOO_000182-T1 and MHACOO_000183-T1, were annotated as PsbP: K02717), photosystem II PsbW protein (PsbW), the enzymes in Calvin-cycle: sedoheptulose-bisphosphatase (SBPase), phosphoribulokinase (PRK), transketolase, ribulose-phosphate 3-epimerase (RPE), malate dehydrogenase (MDH2). Several heat shock proteins (HSPA4, HSPA5, HSP60, HSP90A, HSP90B) and oxidative stress response proteins were also enriched (TXN, BCP, SOD2, DHAR). In addition, many ribosomal proteins were detected, including 21 from the large subunit and 11 from the small subunit. Among the less abundant proteins, several photosynthesis-related components were also detected such as a homolog of photosystem II Psb27 protein (Psb27-like), a homolog of photosystem II 13 kDa protein (Psb28-like), cytochrome c6 (PetJ), Light-harvesting complex II chlorophyll *a/b* binding protein 1 and 2 (LHCB1/B2; MHACOO_000287-T1 was annotated as both K08912: LHCB1 and K08913: LHCB2). Furthermore, to evaluate photosynthetic protein abundance relative to pigment content, we normalized the amounts of differentially abundant photosynthesis-related proteins to the change in total chlorophyll content. This analysis revealed that the relative abundance of PsbP (MHACOO_000182-T1), PsbW, Psb27-like, Psb28-like, cytochrome c6, and LHCB1/B2 per unit chlorophyll was decreased in *LRG* compared with WT (Fig. S2).

### Altered abundance of proteins related to cell wall and photosynthesis in *TTR*

GO and KEGG pathway analyses were also performed on proteins that were significantly more or less abundant in *TTR* compared to WT. No GO terms were significantly enriched. However, KEGG analysis identified 19 annotated proteins (9 more abundant, 10 less abundant; Tables 1, 2). Among the more abundant proteins, several were associated with cell motility and cell wall remodeling, including a motor protein, dynein axonemal heavy chain (DNAH), expansin, mannan endo-1,4-beta-mannosidase (MAN), beta-mannosidase (MANBA). Among the less abundant proteins, photosynthesis-related proteins such as Psb 28-like and light-harvesting complex I chlorophyll *a/b* binding protein 3 (LHCA3) were detected.

**Table 1:** Proteins more abundant in *TTR* compared to WT, annotated with K number

Protein ID	K number	Name	Log2 fold change
MHACOO_006988-T1	K19355	MAN; mannan endo-1,4-beta-mannosidase [EC:3.2.1.78]	4.2
MHACOO_006997-T1	K01192	MANBA, manB; beta-mannosidase [EC:3.2.1.25]	4.1
MHACOO_000003-T1	K10408	DNAH; dynein axonemal heavy chain	2.7
MHACOO_006313-T1	K19355	MAN; mannan endo-1,4-beta-mannosidase [EC:3.2.1.78]	2.5
MHACOO_001334-T1	K03146	THI4, THI1; cysteine-dependent adenosine diphosphate thiazole synthase [EC:2.4.2.60]	2.4
MHACOO_006339-T1	K20628	exlX; expansin	2.3
MHACOO_006996-T1	K01187, K15925	K01187: malZ; alpha-glucosidase [EC:3.2.1.20], K15925: XYL1; alpha-D-xyloside xylohydrolase [EC:3.2.1.177]	1.9
MHACOO_002258-T1	K01895	ACSS1_2, acs; acetyl-CoA synthetase [EC:6.2.1.1]	1.8
MHACOO_007157-T1	K09487	HSP90B, TRA1; heat shock protein 90 kDa beta	1.7

**Table 2:** Proteins less abundant in *TTR* compared to WT, annotated with K number

Protein ID	K number	Name	Log2 fold change
MHACOO_003246-T1	K02437	gcvH, GCSH; glycine cleavage system H protein	− 2.9
MHACOO_006354-T1	K02267	COX6B; cytochrome c oxidase subunit 6b	− 2.9
MHACOO_000646-T1	K01188	beta-glucosidase [EC:3.2.1.21]	− 2.8
MHACOO_001430-T1	K03252	EIF3C; translation initiation factor 3 subunit C	− 2.7
MHACOO_000870-T1	K03946	NDUFA2; NADH dehydrogenase (ubiquinone) 1 alpha subcomplex subunit 2	− 2.7
MHACOO_000275-T1	K08903	psb28; photosystem II 13 kDa protein	− 2.5
MHACOO_002722-T1	K17892	FTRC; ferredoxin-thioredoxin reductase catalytic chain [EC:1.8.7.2]	− 2.2
MHACOO_004418-T1	K01868	TARS, thrS; threonyl-tRNA synthetase [EC:6.1.1.3]	− 2.1
MHACOO_004122-T1	K07178	RIOK1; RIO kinase 1 [EC:2.7.11.1]	− 1.9
MHACOO_004558-T1	K08909	LHCA3; light-harvesting complex I chlorophyll a/b binding protein 3	− 1.5

### Mutant strains exhibit reduced photosynthetic efficiency and slower growth

**Fig. 6:**
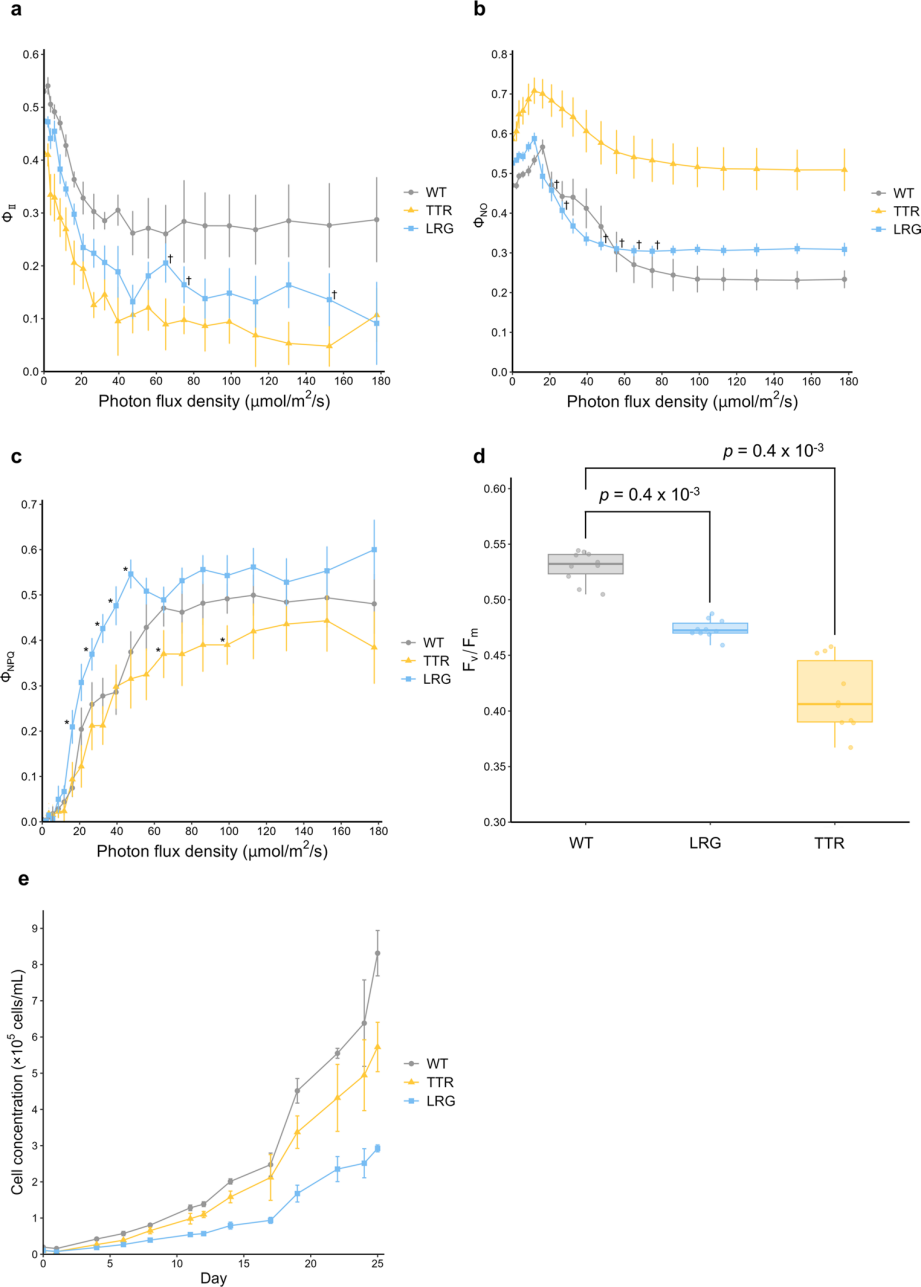
Photosynthetic parameters and cell growth rates of the mutants. (a, b, c) Photosynthetic quantum yield (ΦII), quantum yield of nonregulated energy dissipation (ΦNO), and quantum yield of regulated energy dissipation (ΦNPQ). † in (a, b): not significantly different from WT (*p* > 0.05). All other points are significantly different (*p*< 0.05). * in (c): significantly different from WT (*p* < 0.05). Data are presented as mean ± SD (*n* = 10). (d) Maximum quantum yield (Fv/Fm). Each point represents one AOI measured by PAM. *n* = 10. (e) Cell growth of mutants and WT. Data are presented as mean ± SD (*n* = 3). The *p*-value was calculated using Wilcoxon rank sum test with Bonferroni correction.

Because both mutants showed altered abundances of photosynthesis-related proteins, we measured the photosynthetic quantum yield (Φ_Ⅱ_) (Fig. 6a). Both *LRG* and *TTR* exhibited significantly lower Φ_Ⅱ_ than WT across a wide range of photon flux densities. To further investigate the causes of reduced Φ_Ⅱ_, we measured the quantum yield of nonregulated energy dissipation (Φ_NO_), the quantum yield of regulated energy dissipation (Φ_NPQ_), and the maximum quantum yield (Fv/Fm) (Fig. 6b–d). Both mutants displayed significantly higher Φ_NO_ compared with WT. By contrast, Φ_NPQ_ was significantly higher in *LRG* under low to moderate light intensities (16.1 and 26.6–47.4 μmol m⁻^2^ s⁻^1^), whereas *TTR* generally exhibited lower Φ_NPQ_ than WT. Fv/Fm was significantly reduced in both mutants. In addition, growth rate measurements revealed that both *LRG* and *TTR* grew more slowly than WT (Fig. 6e).

## Discussion

### Increase in cell size is associated with disruption of photosynthetic homeostasis

Photosynthesis is closely related to algal cell proliferation and cell size, but the mechanism has remained unknown (Malerba et al. 2018). The principle that explains why many photosynthetic parameters correlate with cell size is known as the “package effect” (Huan et al. 2022; Malerba et al. 2018). Specifically, this effect describes how, under otherwise identical conditions, the efficiency with which pigment molecules capture photons decreases as cell volume increases (Malerba et al. 2018). In *Nannochloropsis* mutant strains, a correlation has been reported among decreased photosynthetic activity (Φ_Ⅱ_), reduced proliferation rate, and increased cell size (Xu et al. 2023). *LRG* in this study demonstrated that *M. hakoo* exhibited a similar correlation between Φ_Ⅱ_ change and cell size. Taken together, these findings suggest that increasing the cell size of microalgae does not necessarily lead to enhanced photosynthetic capacity.

However, the decrease in Φ_Ⅱ_ observed in *LRG* was also evident under low-light conditions, where the contribution of the package effect is considered to be minimal. Furthermore, *LRG* exhibited reduced Fv/Fm, a parameter representing maximum quantum yield in the dark. These results suggest that, in addition to reduced pigment efficiency, other factors may have suppressed photosynthetic electron transport in *LRG*. Indeed, *LRG* showed an increase in chlorophyll content and fluctuations in the levels of many photosynthesis-related proteins. Intriguingly, however, the abundances of all photosynthetic proteins were lower in *LRG* than in WT when normalized per unit chlorophyll. In particular, the relative abundances of Psb27-like and Psb28-like proteins—components of PSII involved in its assembly and repair (Komenda et al. 2024)—were decreased, suggesting that photoinhibition due to functional impairment of PSII may have contributed to the observed reductions in Fv/Fm and Φ_II_, as well as the increase in Φ_NO_.

These findings indicate that enhancement of photosynthetic electron transport is not simply achieved by increasing the absolute abundance of its components. Instead, the quantitative balance between chlorophyll content and photosynthetic protein abundance, and more broadly among molecules involved in photochemical reactions, appears to be critical for maintaining photosynthetic regulation. Additionally, *LRG* exhibited increased levels of multiple oxidative stress–related proteins, heat shock proteins, and ribosomal proteins, suggesting that disruption of photosynthetic homeostasis placed the cells under stress. The observed upregulation of ribosomal proteins may reflect an enhanced capacity for protein synthesis as part of a stress response to environmental changes within the cell. Although the precise mechanisms remain unclear, the elevated Φ_NPQ_ under low-light conditions in *LRG* may represent an indirect consequence of such stress responses. Overall, these large-scale proteomic changes and impaired photosynthetic performance likely underlie the reduced growth rate observed in *LRG*.

### Cell cycle delay or disruption of cell wall regulation may affect cell morphology

*M. hakoo* is known to divide during the dark phase when cultured in the light–dark cycle (Kato et al. 2023). In contrast, *TTR* obtained in this study showed no significant change in the proportion of mitotic cells regardless of the light and dark cycles, suggesting a potential defect in cell cycle. Proteomic analysis revealed an increased abundance of dynein, a motor protein, in *TTR*. Dynein plays a critical role in spindle formation during cell division (Roberts et al. 2013), and its overexpression has been reported to disrupt microtubule and centrosome organization (King et al. 2003). These findings suggest that excessive dynein levels may have impaired proper cell cycle progression in *TTR*, potentially leading to its reduced growth rate.

In green algae of the class Trebouxiophyceae, such as *Chlorella vulgaris* and *Parachlorella kessleri*, cell division involves the formation of cell wall–derived autospores, making coordinated regulation of cell wall structure and cytoplasmic division essential for successful mitosis (Yamamoto et al. 2004, 2005). In this study, *TTR* exhibited deformed cell walls, increased abundances of cell wall–related proteins (expansin, MAN, and MANBA), and strong cell aggregation. Expansin is a cell wall–loosening protein that disrupts hydrogen bonds within the wall matrix, and its overexpression in the green alga *Micrasterias denticulata* has been shown to cause substantial alterations in cell morphology (Vannerum et al. 2011). In *Chlamydomonas reinhardtii*, high-light treatment induces the formation of multicellular aggregates known as “palmelloid,” accompanied by increased abundance of expansin-like proteins (Suwannachuen et al. 2023). Similarly, MAN and MANBA, whose levels were elevated in *TTR*, are involved in the degradation and remodeling of plant and algal cell walls (Schröder et al. 2009; Spain et al. 2021). Furthermore, mutants in the dual-specificity tyrosine phosphorylation-regulated kinase (DYRKP1) and matrix metalloproteinases (MMPs)—both required for cell wall degradation during cell division—in *C. reinhardtii* have been reported to result in palmelloid formation (Kim et al. 2024). Taken together, these findings suggest that in *TTR*, disrupted regulation of cell wall structure may have increased cell adhesion or prevented daughter cells from being released from autospores, thereby leading to aggregate formation.

In addition, *TTR* exhibited reduced photosynthetic quantum yield and elevated nonregulated energy dissipation, which correlated with decreased levels of Psb28-like and LHCA3 proteins. Because Psb28-like is a component of PSII and LHCA3 is an antenna protein of PSI (Iwai et al. 2024), these changes suggest that electron transfer from PSII to PSI may have been impaired in *TTR*. Although further analyses are required, it is notable that the decreases in Φ_Ⅱ_ and increases in Φ_NO_ in *TTR* were greater than those observed in *LRG*. This raises the possibility that, in addition to changes in photosynthetic proteins, defects in cell cycle progression and alterations in cell wall structure may also have indirectly contributed to the impaired photosynthetic activity of *TTR*.

### Relationship between genome mutations and phenotypes

The types of genomic mutations induced by heavy-ion beam irradiation are known to depend on the linear energy transfer (LET) of the beam. Studies in *Arabidopsis thaliana* and *Oryza sativa* L (rice) have shown that relatively low LET values (23–70 keV μm⁻^1^) tend to generate point mutations and small deletions of less than 100 bp, whereas higher LET values (e.g., 290 keV μm⁻^1^) often result in chromosomal rearrangements and large deletions exceeding 100 bp (Kazama et al. 2011, 2017; Morita et al. 2021). In this study, the irradiation was performed at an LET of 23 keV μm⁻^1^; therefore, the mutations present in the isolated mutants are likely to be point mutations or small deletions.

Because heavy-ion beam irradiation introduces multiple mutations randomly throughout the genome, the phenotypes observed in this study (*LRG*: increased cell size, elevated chlorophyll content, and reduced photosynthetic electron transport; *TTR*: increased proportion of four-cell–like stages and reduced photosynthetic electron transport) are unlikely to result from a single mutation. Instead, they may be attributable to a combination of independently occurring mutations. Future genomic analyses will be required to identify the specific mutation sites. Moreover, the establishment of genetic transformation systems enabling complementation experiments would allow a more detailed elucidation of the relationship between the genomic mutations and the observed phenotypes.

## Conclusion

In this study, we successfully generated two mutants of *M. hakoo* by heavy-ion beam irradiation and demonstrated a clear relationship between alterations in cell morphology and physiological states. Our results also highlight the utility of heavy-ion beam irradiation as a powerful tool for dissecting cellular and physiological mechanisms in microalgae. Although this study focused on the initial characterization of a small number of mutants, obtaining a larger collection of mutants with similar morphological traits, followed by large-scale screening using techniques such as flow cytometry and subsequent phenotypic grouping, could enable a more comprehensive understanding of the links between cell morphology and physiology in algae. 

## Supplementary Information

Below is the link to the electronic supplementary material.

Supplementary Figures

Supplementary Tables

## Data Availability

The proteome data have been deposited in jPOSTrepo (Okuda et al. 2025) (Accession number: JPST004178). All other data are available from the corresponding author.
