# Supplementary material for: Heavy-ion beam-induced mutants of Medakamo hakoo indicate potential associations between photosynthesis and cell size, cell cycle, and cell wall morphology
Source: J Plant Res. 2025 Dec 10;139(1):119–32. doi: 10.1007/s10265-025-01680-2 (PMC12868012; doi:10.1007/s10265-025-01680-2)
Supplement: Supplementary file 1 — Supplementary Figures [file 10265_2025_1680_MOESM1_ESM.pdf]

Supplementary Figures

Article title:
Heavy-ion beam-induced mutants of *Medakamo hakoo* indicate potential associations between photosynthesis and cell size, cell cycle, and cell wall morphology

Journal name:
Journal of Plant Research

Authors:
Yoji Okabe, Yayoi Tsujimoto-Inui, Shinichiro Maruyama, Kazuhide Tsuneizumi, Tsuyoshi Takeshita, Mayuko Sato, Kiminori Toyooka, Tomoko Abe and Sachihiro Matsunaga

Affiliation and e-mail address of the corresponding author:
Graduate School of Frontier Sciences, The University of Tokyo, Chiba, Japan.
sachi@edu.k.u-tokyo.ac.jp

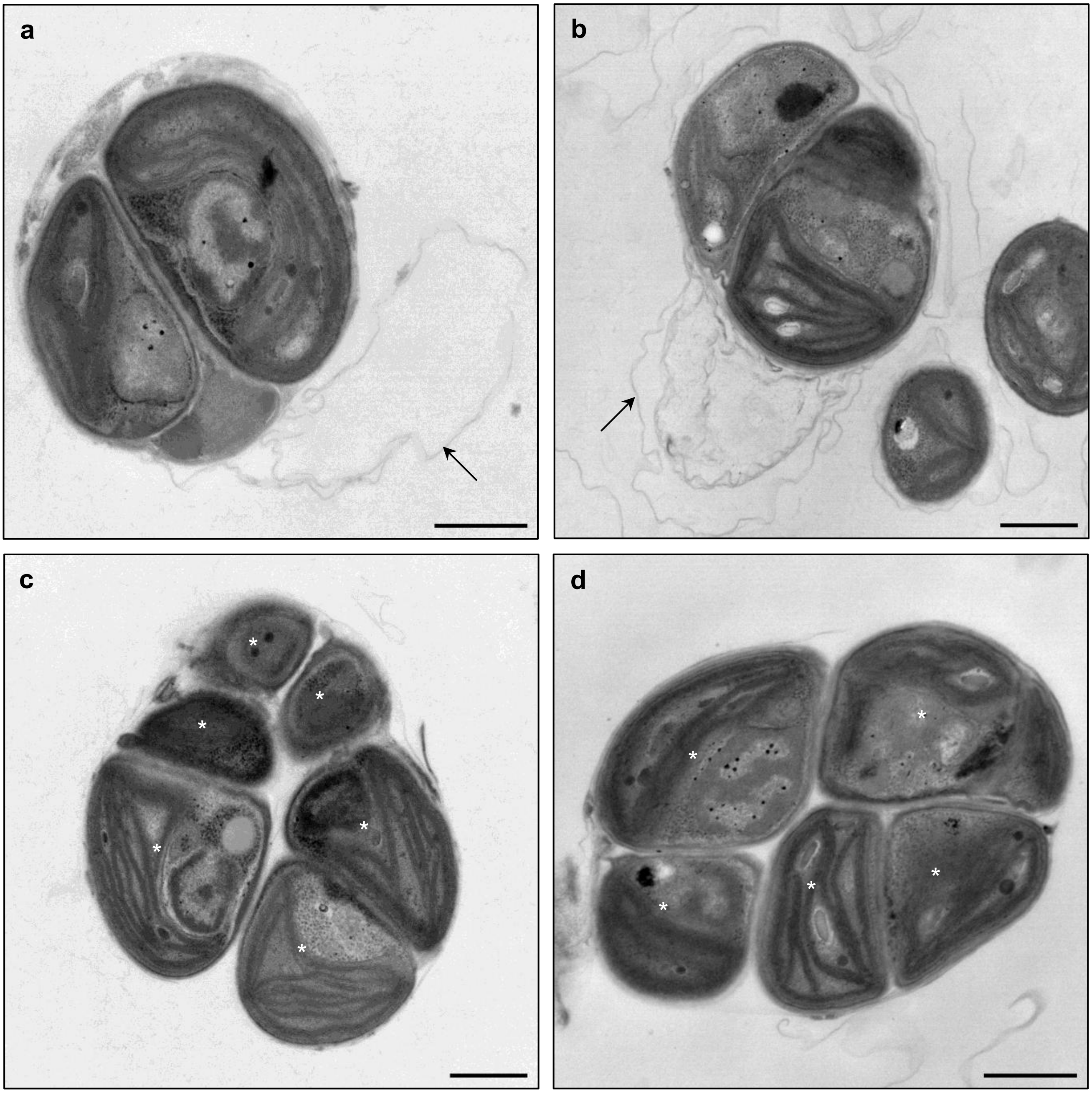

Fig. S1 Cellular structures of the *TTR* mutant. (a, b) Cells with deformed cell walls (arrows). (c, d) Cell aggregates. Asterisks indicate individual cells within each aggregate. (c) An aggregate consisting of six cells. (d) An aggregate consisting of five cells. Scale bars: 500 nm

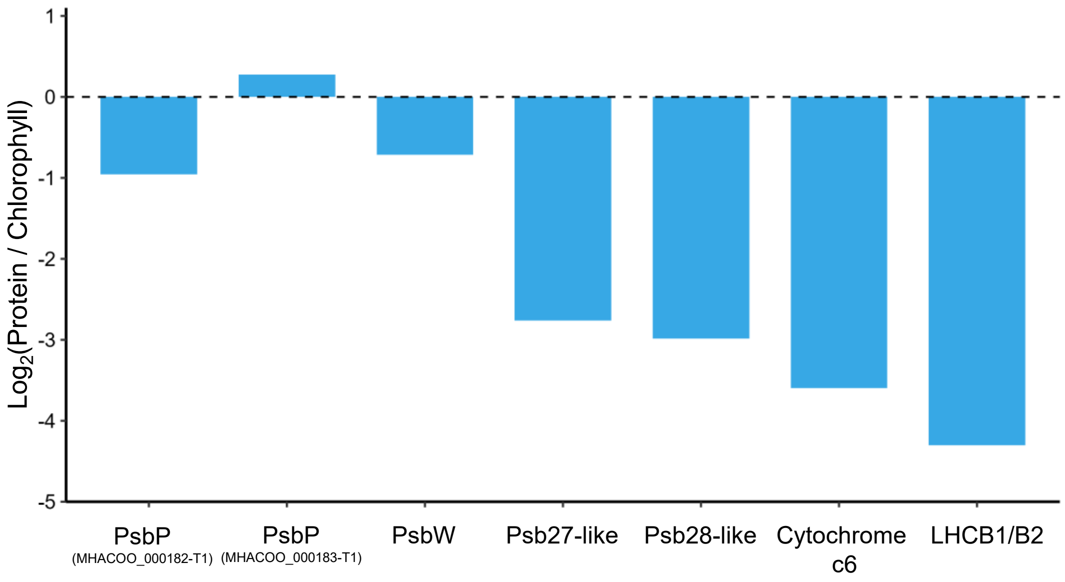

Fig. S2 Changes in photosynthetic proteins per chlorophyll that were significantly altered in *LRG*. The values represent the log2 ratio of the mean fold change of each protein abundance obtained from proteomic analysis to the mean fold change of total chlorophyll amount. PsbP: According to KEGG annotation, two peptide sequences (Protein ID: MHACOO_000182-T1 and MHACOO_000183-T1) were both annotated as PsbP (K02717), and therefore both are shown. LHCB1/B2: A single peptide sequence was annotated as both LHCB1 (K08912) and LHCB2 (K08913). The correspondence between Protein IDs and K numbers is also provided in Tables S2 and S3.
